# The Dementia Literacy Assessment (DeLA): A Novel Measure of Alzheimer's Disease and Related Disorders Health Literacy in Diverse Populations

**DOI:** 10.1002/alz70858_099856

**Published:** 2025-12-24

**Authors:** James E Galvin, Deborah M Germaine, Claudia P Moore, Jennifer A Jeanty, Vaatausili Tofaeono, Lisa Ann Kirk Wiese

**Affiliations:** ^1^ University of Miami Miller School of Medicine, Boca Raton, FL, USA; ^2^ American Samoa Community Cancer Coalition, Pago Pago, American Samoa; ^3^ C.E. Lynn College of Nursing, Florida Atlantic University, Boca Raton, FL, USA

## Abstract

**Background:**

Over the next 20 years, the number of people over 65 and 85 years old is expected to grow by 62% and 84%, respectively.^1^ The older adult population in the US is also becoming more diverse. By 2050, the proportion of older Americans from minoritized populations will increase to 39% with approximately 14 million Hispanics, 8.6 million African Americans, and 5.8 million adults from other ethnoracial groups.^2^ Low health literacy about Alzheimer's disease and related disorders (ADRD) may limit help‐seeking, early detection, and enrollment in clinical trials, particularly in minoritized communities.^3,4,5^ We created the Dementia Literacy Assessment (DeLA) to improve ADRD health literacy.

**Method:**

The DeLA, a story telling method that included two culturally adaptable vignettes embedded with important factoids about Alzheimer's disease (Grandma's Story) and vascular dementia (Hank's Story), was administered to 213 participants from urban and rural regions of South Florida and 193 participants in American Samoa. Inclusion criteria were age 50+, English (US) or Samoan (American Samoa) speaking, and community‐dwelling. Pre‐ and post‐tests were collected to show gains in ADRD health literacy and DeLA results were correlated with objective measures of cognitive performance.

**Result:**

The sample had a mean age of 66.5 ± 9.0y (Range 50‐93), a mean education of 11.8 ± 2.8y (Range 0‐20), and 15.7% reported a family history of AD or another dementia. The sample was 63.4% female and the ethnoracial make‐up was 11.8% White, 39.8% Black or African American, and 48.4% Pacific Islander (predominantly Samoan). The DeLA increased dementia health literacy and performed well across different participant characteristics (age, sex, education, geographic locale, race, ethnicity, cognitive performance) with different ethnoracial and sociodemographic groups demonstrating differential gains in knowledge. Gains in ADRD health literacy were associated with older age, more education, better socioeconomic status, greater resilience, and better cognitive performance.

**Conclusion:**

We demonstrated that dementia health literacy could be increased using a story telling method that included culturally adaptable vignettes embedded with important factoids about ADRD. Increasing ADRD health literacy with the DeLA could increase health‐seeking behaviors in diverse populations for treatment, enrich recruitment into clinical trials, and may help reduce disparities in health outcomes.

